# A Flexible Baseline Measuring System Based on Optics for Airborne DPOS

**DOI:** 10.3390/s21165333

**Published:** 2021-08-07

**Authors:** Yanhong Liu, Wen Ye, Bo Wang

**Affiliations:** 1 Research Institute for Frontier Science, Beihang University, Beijing 100191, China; liu1253891321@163.com (Y.L.); bowang0618@buaa.edu.cn (B.W.); 2 Division of Mechanics and Acoustic Metrology, National Institute of Metrology, Beijing 100029, China

**Keywords:** aerial remote sensing, distributed position and orientation system, flexible baseline measurement

## Abstract

Three-dimensional imaging for multi-node interferometric synthetic aperture radar (InSAR) or multi-task imaging sensors has become the prevailing trend in the field of aerial remote sensing, which requires multi-node motion information to carry out the motion compensation. A distributed position and orientation system (DPOS) can provide multi-node motion information for InSAR by transfer alignment technology. However, due to wing deformation, the relative spatial relationship between the nodes will change, which will lead to lower accuracy of the transfer alignment. As a result, the flexible baseline between the nodes affects the interferometric phase error compensation and further deteriorates the imaging quality. This paper proposes a flexible baseline measuring system based on optics, which achieves non-connect measurement and overcomes the problem that it is difficult to build an accurate wing deformation model. An accuracy test was conducted in the laboratory, and results showed that the measurement accuracy of the baseline under static and dynamic conditions was less than 0.3 mm and 0.67 mm, respectively.

## 1. Introduction

Airborne Synthetic Aperture Radar (SAR) requires a plane to move in a straight line at a constant speed, which is difficult to attain because of external interference such as gust, turbulence, and engine vibration. The position and orientation system (POS) can provide high-precision motion information for SAR to compensate for its motion error and then realize two-dimensional imaging with high resolution [1]. With the development of an airborne earth observation system, three-dimensional imaging for multi-node interferometric synthetic aperture radar (InSAR) or multi-task imaging sensors has become the prevailing trend [2,3,4], which requires multi-node motion information to carry out the motion compensation. A single POS cannot achieve the measurement of multi-node motion information. Therefore, a distributed position and orientation system (DPOS) needs to be developed. DPOS mainly includes a main POS, a few sub-IMUs, and a distributed POS computer system (DPCS). The main POS integrates a high-precision inertial measurement unit (IMU) and global navigation satellite system (GNSS). The sub-IMU only consists of a low-precision IMU [1].

**Figure 1:**
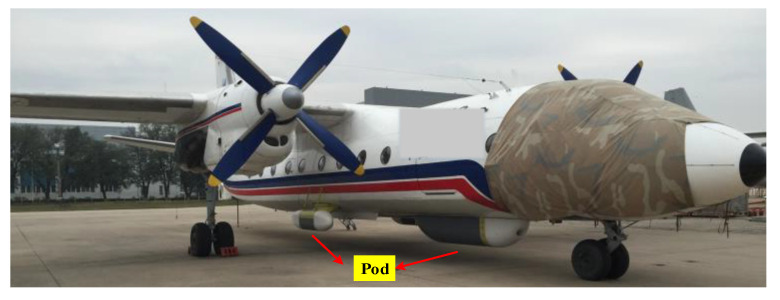
The installation layout of SAR antennas (in the pod below the belly).

**Figure 2:**
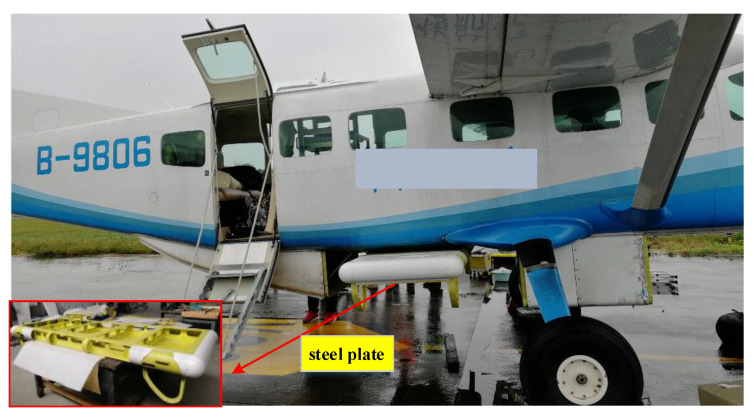
The installation layout of SAR antennas (inside the steel plate).

Analyzed from the three-dimensional imaging principle of interferometric SAR (InSAR) or array SAR, the longer the baselines between multiple nodes are the higher the three-dimensional imaging accuracy is. In general, SAR antennas are installed in the pod below the belly, as shown in Figure 1. In order to increase baseline length, the aircraft is refitted by attaching a steel plate and SAR antennas are located inside the steel plate, as shown in Figure 2. In order to increase the baseline length further and pursue higher imaging accuracy, installing several radar pods on the wings is under consideration.

**Figure 3:**
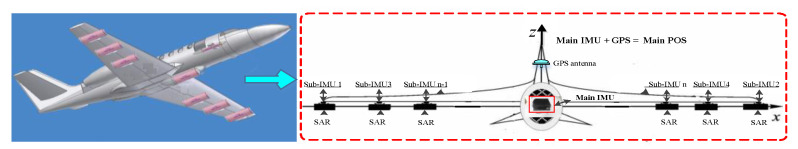
The installation layout of DPOS.

In general, the main POS is installed in the belly, and a few sub-IMUs are installed on the wing near the phase center of the SAR. Figure 3 shows the installation layout of DPOS. The high-precision motion information of the main POS is provided for each sub-IMU as reference and high-accuracy motion information of each sub-IMU is realized by transfer alignment technology. Due to the wing deformation, the flexible baseline between the main POS and the sub-IMU will seriously degrade the performance of transfer alignment.

Airborne InSAR uses the geometric relationship between radar wavelength, interferometric phase, the aircraft’s height, baseline length, and beam direction to measure the three-dimensional position information of the target on the ground. The radar wavelength and interferometric phase depend on InSAR technology. The aircraft’s height and beam direction are measured in real time by DPOS. Hypothesizing that each SAR is configured with a high-precision POS, the baseline can be calculated directly. However, the measurement accuracy of the baseline cannot meet the requirement of imaging because the accuracy of the single POS is at the centimeter level and measuring the accuracy of the baseline is certainly at the centimeter level. Given the above analysis, it can be seen that the imaging accuracy of InSAR depends mainly on InSAR technology, DPOS, and the measurement accuracy of the baseline. At the same time, the baseline measurement determines the performance of DPOS. Therefore, baseline measurement becomes the core issue for InSAR.

Accurate baseline measurement is mainly determined by an accurate model of wing deformation. Until now, some studies have idealized the wing deformation as a Markov process [5,6,7] and some parameters in the Markov model have been confirmed by experience. Liu et al. use elastic mechanics to simulate the process of wing deformation [8]. However, the wing deformation model established by this method varies for different aircraft material, which lacks practicality.

Due to the advantages of non-contact, fast speed, and high precision, optical measurement has been widely applied in many fields [9,10,11,12]. For example, among almost all the wind tunnel tests, wing deformation is measured with an optical camera [13]. In this paper, a flexible baseline measuring system for airborne DPOS is proposed. The relative position and orientation between the main POS and the sub-IMU are measured by two cameras, and, further, the flexible baseline measurement can be realized. Taking the distance between the main POS and sub-IMUs into account, there are non-overlapping fields of view between cameras. In this paper, the hand–eye calibration method [14,15,16] is used to solve the external parameters between cameras with non-overlapping fields of view.

The biggest advantage of a flexible baseline measuring system is that it can directly measure wing deformation information and further achieve baseline measurement. In addition, the measurement accuracy of the baseline can be gradually enhanced by improving the vision algorithm. Higher measurement accuracy of the baseline will directly improve the imaging performance of the InSAR. In addition, it can also improve the performance of DPOS by transfer alignment technology, which indirectly improves the imaging performance of the InSAR.

## 2. System Overview and External Parameter Calibration Method for Two Cameras with Non-Overlapping Fields of View

### 2.1. System Overview

**Figure 4:**
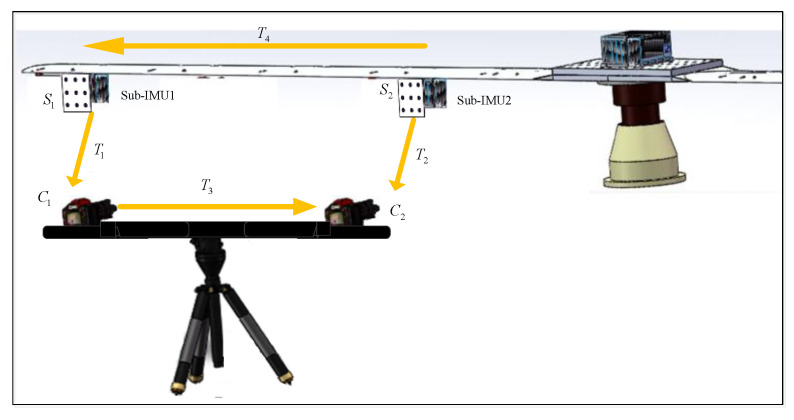
Schematic diagram of the flexible baseline measuring system.

Figure 4 shows the schematic diagram of a flexible baseline measuring system. Two sub-IMUs are installed on the wing, and two targets are attached to the corresponding sub-IMUs’ surfaces. Two cameras installed on a tripod are rigidly linked. For practical applications of airborne DPOS, the distance between the two sub-IMUs is long and there are non-overlapping fields of view between cameras; as a result, camera $C_{1}$ can only “see” the target $S_{1}$, and the camera $C_{2}$ can only “see” the target $S_{2}$.

Variables $T_{1}$ and $T_{2}$ are homogeneous transformation matrices of the target $S_{1}$ relative to the camera $C_{1}$ and the target $S_{2}$ relative to the camera $C_{2}$, both of which can be calculated by the Perspective-n-Point (PnP) method [17,18]. Variable $T_{3}$ is the homogeneous transformation matrix between the two cameras; its calculation process is presented in detail in Section 2.2. 

The homogeneous transformation matrix $T_{i}\left(i=1,2,3,4\right)$ is represented with a rotation matrix $R_{i}$ and a translation vector $t_{i}$ as follows:

(1) $$T_{i}=\left(\begin{array}{cc} R_{i} & t_{i}\\ 0_{1\times 3} & 1 \end{array}\right)\;$$

where $R_{i}$ is a 3×3 rotation matrix, which is represented with three Euler angles around the x-axis, y-axis, and z-axis, respectively. $t_{i}$ is a 3×1 translation vector.

Analyzed from the flexible baseline measuring system, once $T_{1},\;\;T_{2}$, and $T_{3}$ are calculated, the homogeneous transformation matrix $T_{4}$ between the two targets can be calculated easily. Then, the flexible baseline can be recovered between the two targets, which is the final variable to be solved.

### 2.2. External Parameter Calibration Method for the Two Cameras with Non-Overlapping Fields of View

The classical stereo calibration algorithm [19] is not suitable to calibrate external parameters between the two cameras with non-overlapping fields of view. In this paper, the hand–eye calibration method, which originated in robotics, is used to cope with this problem. First, the principle of the hand–eye calibration method in robotics is presented and second, this method is extended to solve the external parameters between cameras with non-overlapping fields of view.

**Figure 5:**
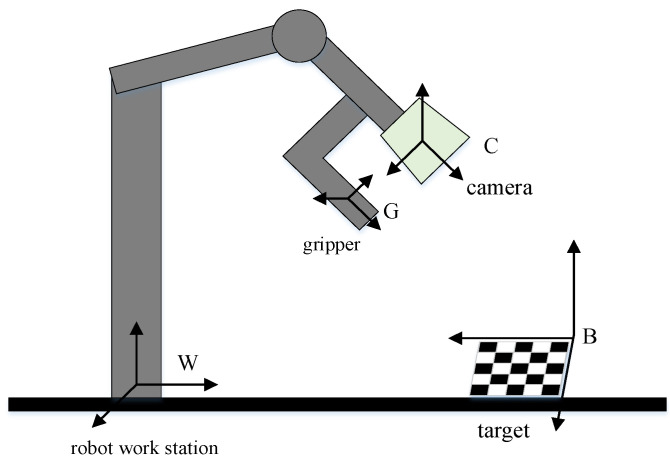
Schematic diagram of hand–eye calibration in robotics.

A schematic diagram of the hand–eye calibration method in robotics is shown in Figure 5. The camera and robot gripper are rigidly connected and the goal is to determine the relative position and orientation between the camera and the robot gripper. For convenience, some coordinate systems used in this paper are defined as follows [20]:

G: the gripper coordinate system, which is fixed on the robot gripper and moves along with the gripper.

C: the camera coordinate system, whose origin is at the camera lens.

B: the target coordinate system, which is fixed on the target.

W: the robot world coordinate system, which is fastened to the robot work station. As the robot arm moves, the encoder output can communicate the position relationship between the gripper and robot work station.

As the gripper moves, the camera remains focused on the target; then the position relationship between camera and gripper can be solved.

Assuming the gripper is replaced with a camera, hand–eye calibration can be used to solve the relative position relationship between two cameras with non-overlapping fields of view.

**Figure 6:**
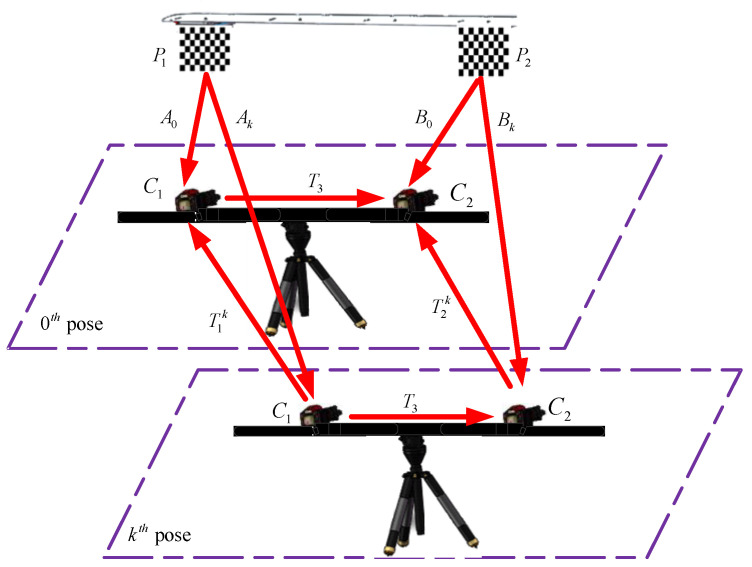
The schematic of the two cameras’ calibration with non-overlapping fields of view.

As shown in Figure 6, the two targets $P_{1}$ and $P_{2}$ are rigidly linked, as are the two cameras $C_{1}\;$ and $C_{2}$. Let the two cameras perform motions K(K≥20) times. Each camera pose is expressed relative to its first pose ($0^{\mathrm{th}}$ pose). $T_{1}^{k}$ denotes the homogeneous transformation of the camera $C_{1}\;$ from $0^{\mathrm{th}}$ pose to $k^{\mathrm{th}}$ pose, for k=1,2,⋯⋯K. Similarly, $T_{2}^{k}$ indicates the homogeneous transformation of the camera $C_{2}$ from $0^{\mathrm{th}}$ pose to $k^{\mathrm{th}}$ pose. $T_{3}$ is the unknown homogeneous transformation between the two cameras.

Based on the above analysis, the calibration process of external parameters for two cameras with non-overlapping fields of view can be summarized in detail as follows:

Step 1: Solve the external parameters of the camera relative to the corresponding target ($A_{0},A_{k},B_{0},B_{k}$).

As shown in Figure 4, let $A_{k}$ represent the external parameters of camera $C_{1}$ relative to target $P_{1}$ at $k^{\mathrm{th}}$ pose. $A_{k}$ consists of the rotation matrix $R_{k}$ and translation vector $t_{k}$, and its expression is shown as Equation (2). $A_{0}$ represents the external parameters of camera $C_{1}$ relative to target $P_{1}$ at $0^{\mathrm{th}}$ pose. All of these can be calculated by using the MATLAB calibration toolbox developed by Zhang’s calibration method [21,22].

(2) $$A_{k}=\left(\begin{array}{cc} R_{{}_{k}} & t_{k}\\ 0_{1\times 3} & 1 \end{array}\right)\;$$

In the same way, let $B_{k}$ represent external parameters of camera $C_{2}$ relative to target $P_{2}$ at $k^{\mathrm{th}}$ pose and $B_{0}$ represent external parameters of camera $C_{2}$ relative to target $P_{2}$ at $0^{\mathrm{th}}$ pose. Referring to the calculation process of $A_{k}$ mentioned above, $B_{0}$ and $B_{k}$ can be calculated by the same method.

Step 2: Solve the camera pose relative to its first pose ($0^{\mathrm{th}}$ pose).

According to rigid body rotation theory, the camera pose relative to its first pose ($0^{\mathrm{th}}$ pose) can be obtained by the homogeneous transformation between camera and target before and after the camera motion. The expression of $T_{1}^{k}$ and $T_{2}^{k}$ can be written as

(3) $$\left\{ \begin{array}{l} T_{1}^{k}=A_{0}A_{k}^{-1}\\ T_{2}^{k}=B_{0}B_{k}^{-1}\; \end{array}\right.$$

Step 3: Solve the homogeneous transformation ($T_{3}$) between the two cameras. 

According to the system overview introduced in Section 2.1, the equation for $T_{3}$ can be derived by

(4) $$T_{2}^{k}T_{3}=T_{3}T_{1}^{k}$$

Equation (4) is the hand–eye calibration model with the form AX=XB, where  X is the unknown matrix to be determined. Further, Equation (4) can be broken down into

(5) $$\{\begin{array}{l} R_{2}^{k}R_{3}=R_{3}R_{1}^{k}\\ R_{2}^{k}t_{3}+t_{2}^{k}=R_{3}t_{1}^{k}+t_{3} \end{array}$$

Equation (5) can easily be solved by linear algebra, but it is a linear homogeneous system that theoretically has an infinite solution. In order to obtain a unique solution, the Lie group and Lie algebra theory [23] are used. 

Rigid-body motions can be expressed by a Euclidean group, which consists of a matrix described by the following form [24]:

(6) $$\left[\begin{array}{cc} R & T\\ 0 & 1 \end{array}\right]$$

where $R\in SO(3),T\in R^{3}$. Here, SO(3) represents a group of rotation matrices. The transformation from Lie algebra to Lie group satisfies the exponential mapping relationship. If [ω]∈SO(3),exp[ω]∈SO(3), then its exponential mapping satisfies the following formula:

(7) $$\{\begin{array}{l} \exp\left[\omega \right]=I+\frac{\sin\left\|\omega \right\|}{\left\|\omega \right\|}\left[\omega \right]+\frac{1-\cos\left\|\omega \right\|}{{\left\|\omega \right\|}^{2}}{\left[\omega \right]}^{2}\\ \left[\begin{array}{ccc} 0 & -\omega_{3} & \omega_{2}\\ \omega_{3} & 0 & -\omega_{1}\\ -\omega_{2} & \omega_{1} & 0 \end{array}\right]\overset{\Delta }{=}\left[\omega \right]\\ {\left\|\omega \right\|}^{2}=\omega_{1}^{2}+\omega_{2}^{2}+\omega_{3}^{2} \end{array}$$

The transformation from Lie group to Lie algebra satisfies the logarithmic mapping relationship. If θ∈SO(3), then its logarithmic mapping can be expressed as follows:

(8) $$\log\theta =\frac{\phi }{2\sin\phi }(\theta -\theta^{T})$$

where ϕ satisfies 1+2cosϕ=tr(θ) and ${\left\|\log\theta \right\|}^{2}=\phi^{2}$. According to Equation (5), $R_{2}^{k}$ can be expressed as

(9) $$R_{2}^{k}=R_{3}R_{1}^{k}R_{3}^{T}$$

Let $\log\;R_{2}^{k}=\left[\alpha^{k}\right]$ and $\log\;R_{1}^{k}=\left[\beta^{k}\right]$, then $R_{2}^{k}=R_{3}R_{1}^{k}R_{3}^{T}$ can be rewritten as:

(10) $$\left[\alpha^{k}\right]=\log(R_{3}R_{1}^{k}R_{3}^{T})=R_{3}\left[\beta^{k}\right]R_{3}^{T}=\left[R_{3}\beta^{k}\right]$$

Then, it yields

(11) $$\alpha^{k}=R_{3}\beta^{k}$$

Now the optimal value of $R_{3}$ can be found by minimizing the following cost function

(12) $$\sum_{k=1}^{K}{\left\|R_{3}\beta^{k}-\alpha^{k}\right\|}^{2}$$

that

(13) $$R_{3}={\left(M^{T}M\right)}^{-\frac{1}{2}}M^{T}$$

where $M=\sum_{k=1}^{K}\beta^{k}{\left(\alpha^{k}\right)}^{T}$. 

Then, by combining Equation (5) with (13), $t_{3}$ can be calculated by

(14) $$t_{3}={\left(C^{T}C\right)}^{-1}C^{T}d$$

where

(15) $$C=\left[\begin{array}{c} R_{2}^{1}-I\\ R_{2}^{2}-I\\ \vdots \\ R_{2}^{K}-I \end{array}\right],d=\left[\begin{array}{c} R_{3}t_{1}^{1}-t_{2}^{1}\\ R_{3}t_{1}^{2}-t_{2}^{2}\\ \vdots \\ R_{3}t_{1}^{K}-t_{2}^{K} \end{array}\right]$$

So far, $R_{3}$ and $t_{3}$ have been calculated. Then $T_{3}$ can be solved with

(16) $$T_{3}=\left(\begin{array}{cc} R_{3} & t_{3}\\ 0_{1\times 3} & 1 \end{array}\right)$$

## 3. Flexible Baseline Measurement

The flexible baseline measuring system proposed in this paper is used to measure flexible baseline between multiple nodes, which are mainly divided into three major steps.

Step 1: Solve the homogeneous transformation ($T_{1}$ and $T_{2}$) between camera and target.

Before solving $T_{1}$ and $T_{2}$, the camera intrinsic parameters must be known, which can be calculated by Equation (2). If the coordinates of each feature point in the world coordinate system (target coordinate system) and the image coordinates of the corresponding feature points are known, the homogeneous transformation ($T_{1}$ and $T_{2}$) between camera c and target can be calculated by the PnP algorithm.

Step 2: Solve the homogeneous transformation ($T_{4}$) between the two targets.

So far, $T_{1},T_{2}$, and $T_{3}$ have been calculated. The homogeneous transformation $T_{4}$ between the two targets can be obtained by Equation (17).

(17) $$T_{4}=T_{2}T_{3}^{-1}T_{1}^{-1}$$

Step 3: Solve the flexible baseline.

**Figure 7:**
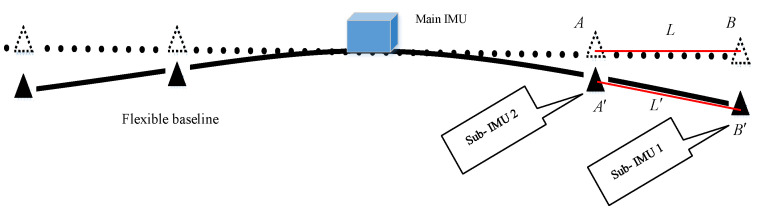
Baseline measurement diagram.

As shown in Figure 7, point A represents sub-IMU2 and point B represents sub-IMU1. L denotes the baseline length between point $A(x_{A},y_{A},z_{A})$ and point $B(x_{B},y_{B},z_{B})$ under the initial condition of the wing without deformation, which is shown with a red line in Figure 6. $L^{\prime }$ denotes the baseline length between point $A^{\prime }(x_{A^{\prime }},y_{A^{\prime }},z_{A^{\prime }})$ and point $B^{\prime }(x_{B^{\prime }},y_{B^{\prime }},z_{B^{\prime }})$ in the case that the wing is subjected to external force and deformed, which can be calculated by the following formula:

(18) $$L^{\prime }=\sqrt{{\left(x_{A^{\prime }}-x_{B^{\prime }}\right)}^{2}+{\left(y_{A^{\prime }}-y_{B^{\prime }}\right)}^{2}+{\left(z_{A^{\prime }}-z_{B^{\prime }}\right)}^{2}}$$

## 4. Laboratory Tests for Flexible Baseline Measurement

### 4.1. External Parameter Calibration Method for the Two Cameras ($T_{3}$)

#### 4.1.1. DPOS Demonstration Platform

**Figure 8:**
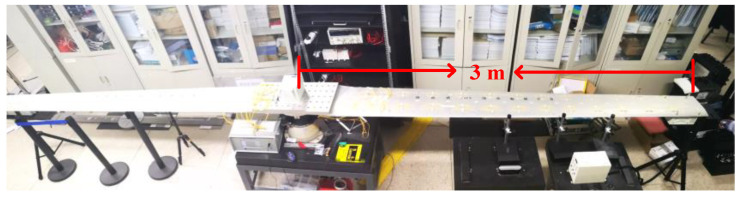
DPOS demonstration platform.

As shown in Figure 8, a DPOS demonstration platform is designed according to the shape and characteristics of the real wing, which is made of aluminum alloy 7075. One side length of the wing is 3 m.

#### 4.1.2. Cameras

**Figure 9:**
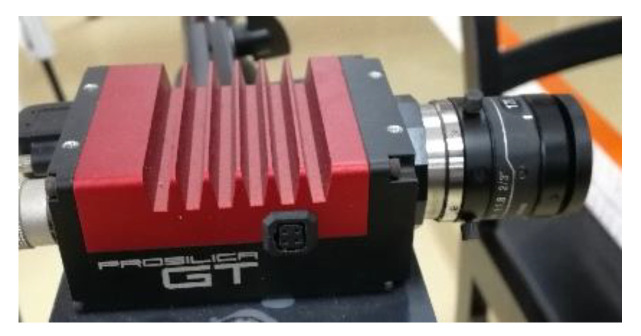
Camera.

The cameras used in the experiment are AVT gt2450, as shown in Figure 9. The camera parameters are shown in Table 1. 

### 4.2. Flexible Baseline Measurement

#### 4.2.1. Static Test

**Figure 10:**
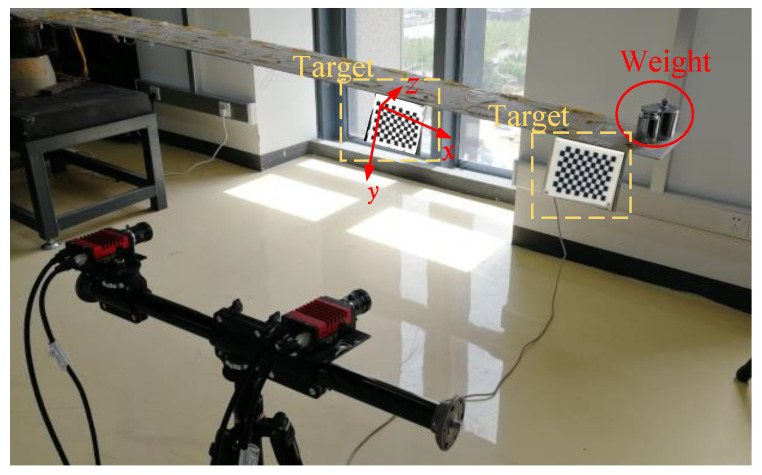
Static test for flexible baseline measurement.

**Figure 11:**
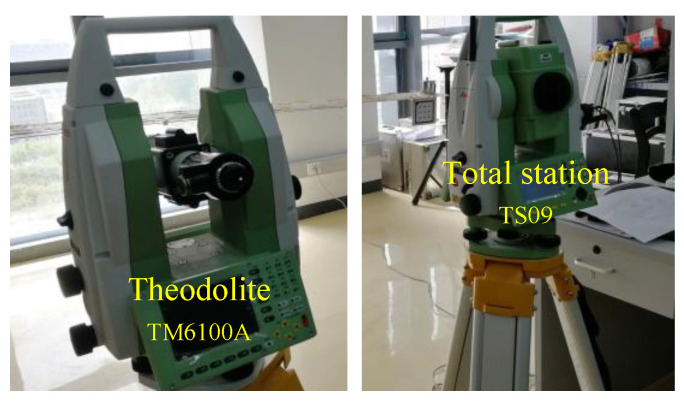
Benchmark system.

The test based on the demonstration platform was carried out. The two targets were placed on the wing where the two adjacent sub-IMUs were mounted, as shown in Figure 10. In this test, the two cameras were placed in front of the two targets with a distance of 1 m. Loads of 1 kg, 2 kg, 3 kg, 4 kg, 5 kg, 6 kg, 7 kg, and 8 kg were added to the wing sequentially. The three-dimensional coordinates measuring system with bino-theodolites (TCMSBT) were taken as the benchmark of flexible baseline measurement, which consists of a theodolite TM6100A and a total station TS09, and its measurement accuracy was up to 0.05 mm, as shown in Figure 11. 

The relative deformation and baseline between the two targets and the baseline error were calculated, and the results are shown in Table 2, from which it can be concluded that the baseline measurement accuracy under static conditions is better than 0.3 mm.

#### 4.2.2. Dynamic Test

**Figure 12:**
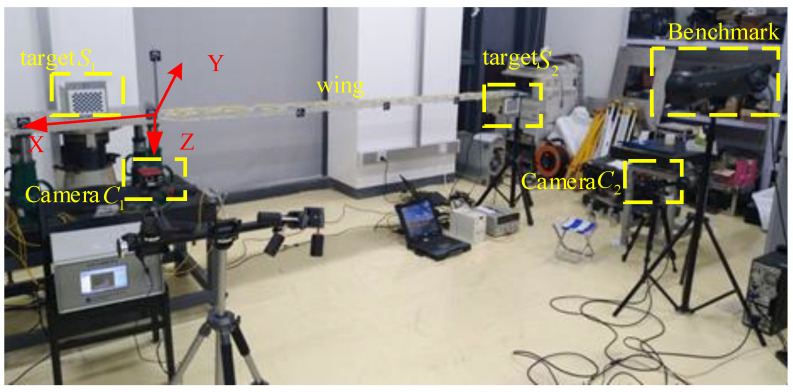
Dynamic test for flexible baseline measurement.

The dynamic test for flexible baseline measurement is shown in Figure 12. An external force was imposed on the end of the wing, and then it was suddenly removed. Next, the wing vibrated up and down freely, which lasted for about 600 s. The high-precision dynamic measuring system developed by Xintuo 3D Technology (Shenzhen) Limited Company was taken as the benchmark, whose accuracy is up to 0.02 mm.

**Figure 13:**
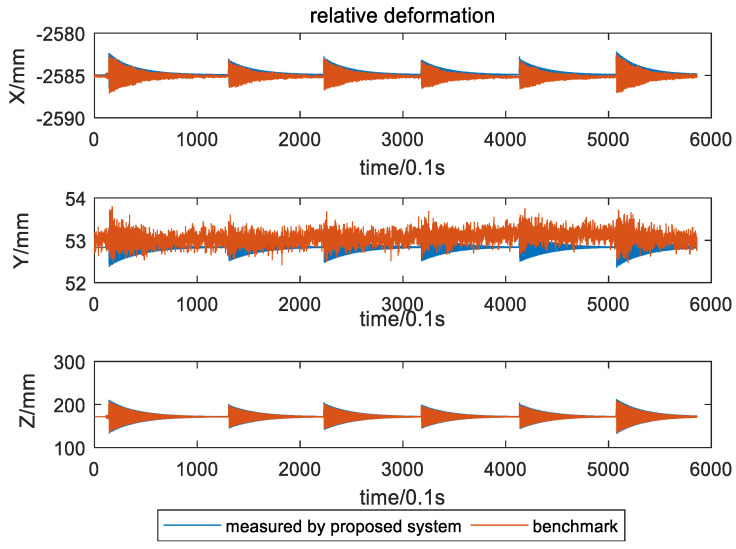
Relative deformation.

**Figure 14:**
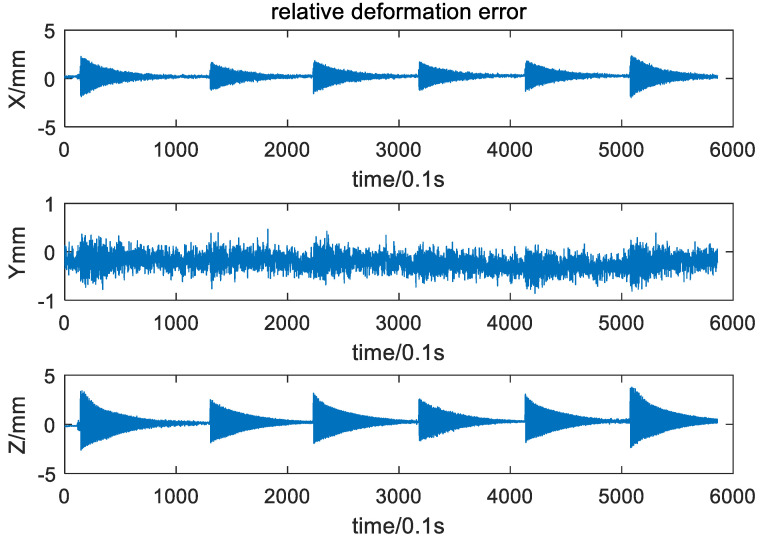
Relative deformation error.

The relative deformation between the two targets and relative deformation error are shown in Figure 13 and Figure 14. It can be seen that the wing showed periodic motion six times with damping amplitude. Therefore, taking the Root Mean Square Error (RMSE) as the error criterion, the relative deformation and its error in the six time periods were calculated, and the results are shown in Table 3. The measurement accuracy of the baseline under dynamic conditions is better than 0.67 mm.

## 5. Conclusions

A flexible baseline measuring system for airborne DPOS has been proposed. Two cameras with non-overlapping fields of view and two targets were utilized to measure the flexible baseline between the nodes. Benchmark tests in a laboratory were conducted and the results showed that the baseline measuring errors under static and dynamic conditions were less than 0.3 mm and 0.67 mm respectively. 

In the future, the experiment will be tested in a real flight environment combining DPOS and InSAR, where the imaging sensors (cameras) are located in a pod below the belly and the targets observed through the pod’s windows. Here, general industrial cameras can all be used in the proposed system. However, the camera is easily disturbed by weather, temperature, and light, which deteriorates the measurement accuracy of the baseline, so a robust algorithm against adverse working conditions will be paid more attention. 

## Figures and Tables

**Table 1:** Camera parameters.

Camera Parameters	Index
Image resolution	2448 * 2050
Frame rate	15 fps
Focal length	25 mm
Size of CCD pixel	3.45 μm * 3.45 μm
lens	Computar M2518-MPW2

**Table 2:** Measurement results (mm).

Loads	x-axis		y-axis		z-axis		Baseline		||Baseline Error||
	Proposed Method	Benchmark	Proposed Method	Benchmark	Proposed Method	Benchmark	Proposed Method	Benchmark	
1 kg	556.875	556.575	6.103	7.756	10.037	10.234	556.999	556.723	0.276
2 kg	558.319	558.43	12.605	12.878	10.366	10.42	558.558	558.676	0.118
3 kg	558.916	559.112	18.043	18.007	10.832	11.231	559.312	559.515	0.203
4 kg	559.105	559.332	25.076	25.865	11.121	11.442	559.778	560.047	0.269
5 kg	559.652	559.452	31.184	31.947	11.348	11.567	560.635	560.483	0.152
6 kg	559.863	559.763	37.219	35.743	11.602	12.012	561.219	561.032	0.187
7 kg	560.479	560.7	43.292	43.931	11.731	12.321	562.271	562.553	0.282
8 kg	561.516	561.23	49.619	49.419	11.894	12.305	563.83	563.536	0.294

**Table 3:** Relative deformation and relative deformation error (RMSE: mm).

Time Periods	x-axis	y-axis	z-axis	Baseline Error
0–100 s	0.57	0.25	0.86	0.61
101–200 s	0.43	0.23	0.66	0.45
201–300 s	0.48	0.25	0.76	0.51
301–400 s	0.46	0.30	0.70	0.45
401–500 s	0.49	0.35	0.74	0.51
501–586 s	0.64	0.28	1.01	0.67

## Data Availability

The data presented in this study are available on request from the corresponding author.
